# Does Adjuvant Treatment With Ginkgo Biloba to Statins Have Additional Benefits in Patients With Dyslipidemia?

**DOI:** 10.3389/fphar.2018.00659

**Published:** 2018-06-22

**Authors:** Yu Fan, Xin Jin, Changfeng Man, Dandan Gong

**Affiliations:** Institute of Molecular Biology and Translational Medicine, The Affiliated People’s Hospital, Jiangsu University, Zhenjiang, China

**Keywords:** ginkgo biloba, statins, dyslipidemia, randomized controlled trials, meta-analysis

## Abstract

**Objective:** Ginkgo biloba are widely used alone or in combination with other lipid-lowering agents in the treatment of dyslipidemia in China. We conducted this meta-analysis to investigate whether adjuvant treatment with ginkgo biloba leaves to statins has incremental benefits in patients with dyslipidemia.

**Methods:** Potential studies were searched from PubMed, EMBASE, Cochrane Library, China National Knowledge Infrastructure, VIP, and Wanfang database up to October 2017. Only randomized controlled trials (RCTs) comparing the efficacy and safety of ginkgo biloba leaves plus statins versus statins alone in patients with dyslipidemia were included.

**Results:** Eight RCTs involving 664 patients were included. Compared with statins therapy alone, combination of statins and ginkgo biloba leaves therapy achieved greater reductions in triglycerides [mean difference (MD) -0.32 mmol/L; 95% confidence interval (CI) -0.43 to -0.20], total cholesterol (MD -0.61 mmol/L; 95% CI -0.90 to -0.33), or low-density lipoprotein cholesterol (LDL-C) (MD -0.32 mmol/L; 95% CI -0.48 to -0.16), and a greater increment in high-density lipoprotein cholesterol (MD 0.26 mmol/L; 95% CI 0.15 to 0.37). Subgroup analyses showed that ginkgo biloba leaves plus simvastatin appeared to achieve a greater reduction in serum levels of triglycerides, total cholesterol, and LDL-C than in combination with atorvastatin therapy.

**Conclusion:** This meta-analysis suggests that adjuvant treatment with ginkgo biloba leaves appears to improve blood lipid parameters than statins therapy alone. More well-designed RCTs are needed to investigate the benefits of the combination of statins and ginkgo biloba leaves.

## Introduction

Cardiovascular diseases are still the major cause of mortality in the world (Roger et al., 2012). Dyslipidemia is a well-known contributor to cardiovascular disease (Toth et al., 2012; Roh et al., 2013; Aekplakorn et al., 2014). Approximately 41.9% adults were diagnosis of dyslipidemia in China (Huang et al., 2014). Despite various lipid-modifying therapeutic approaches, overall 38.5% of those individuals did not reach the recommended goal for low-density lipoprotein cholesterol (LDL-C) (Zhao et al., 2014). Therefore, new therapeutic approaches to treat dyslipidemia are an urgent need.

Statins are the forefront of recommended therapies for target LDL-C reduction (Zoungas et al., 2014). However, elevated levels of triglycerides (TG) and low levels of high-density lipoprotein cholesterol (HDL-C) may not be adequately controlled with statin monotherapy. Treatment combining a statin with other lipid-modifying drugs is required for the mixed hyperlipidemia. Along with long-term use of statins in combination with other lipid-modifying agents, incremental adverse effects including transaminase or creatine kinase elevation and skeletal muscle pain are the most common concerns (Choi and Shin, 2014).

Traditional Chinese medicine (TCM) is widely used to treat dyslipidemia (Guo et al., 2014). There are more than 50 herbs or herbal formulas have been used to treat hyperlipidemia (Xie et al., 2012). Leaf extract of Ginkgo biloba is increasingly used as an herbal medicine for treatment of neurodegenerative, cardiovascular, and cerebrovascular diseases (Mohanta et al., 2014). The standardized extract of Ginkgo biloba leaves (EGb 761) contain mainly 24% flavonoid glycosides (24%), 7% proanthocyanidins, and 7% proanthocyanidins, and organic acids of low molecular weight (DeFeudis et al., 2003; Chan et al., 2007). Many studies (Zhou, 2010; Guo and Ju, 2011; Lin et al., 2011; Zhong and Bao, 2011; Jin et al., 2012; Ren, 2012; Zhu, 2012; Xu, 2013) have suggested that leaves of Gingko biloba had therapeutic potential to improve dyslipidemia. However, whether statins combined with ginkgo biloba has synergistic effects on serum lipids improvement is largely unknown. This meta-analysis summarized the available findings from randomized controlled trials (RCTs) to investigate the incremental benefits of gingko biloba leaves as an adjunctive therapy to statins for the management of dyslipidemia.

## Materials and Methods

### Literature Search

This meta-analysis was conducted in accordance with the Preferred Reporting Items for Systematic Reviews and Meta-Analyses (PRISMA) guidelines (Moher et al., 2009). Potential studies were searched from PubMed, EMBASE, Cochrane Library, China National Knowledge Infrastructure, VIP, and Wanfang database up to October 2017. The following medical subject headings were used in the literature search: “gingko biloba” AND “statins” OR “hydroxymethylglutaryl- CoA reductase inhibitors” OR “lovastatin” OR “fluvastatin” OR “simvastatin” OR “pravastatin” OR “atorvastatin” OR” rosuvastatin” OR “pitavastatin” AND “dyslipidemias” OR “hyperlipidemias.” Only papers issued in English and Chinese were included. All references of the selected trials were examined manually to identify additional eligible papers.

### Inclusion and Exclusion Criteria

Studies meeting the following criteria were included: (1) RCTs comparing the effectiveness and safety of ginkgo biloba leaves plus statins versus statins alone in patients with dyslipidemia; and (2) reporting serum levels of LDL-C, HDL-C, TG, or total cholesterol (TC). Studies were excluded if: (1) ginkgo biloba leaves in combination with other Chinese herbal preparations as the intervention; (2) apart from the ginkgo biloba leaves, different regimens have been used between groups; and (3) studies investigating ginkgo biloba leaves versus placebo or other lipid-modifying agents.

### Data Extraction and Quality Assessment

The following data were independently extracted by two reviewers: first author’s surname, publication year, sample size, mean age or range of participants, ratio of men/women, diagnostic criteria of dyslipidemia, intervention (regimen and period), lipid parameters (TC, TG, LDL-C, HDL-C), and adverse events. The methodological quality of included trials was evaluated using Cochrane risk of bias tool of RCTs. If trial met all the criteria, the trial was judged to have low risk of bias; if one or more criteria not met, the trial was judged to have high risk of bias. If insufficient data judge risk of bias, the trial was considered as unclear risk.

### Statistical Analysis

Serum lipid parameters were expressed as continuous data and calculated as mean difference (MD) with the 95% confidence interval (CI). Heterogeneity across the studies was assessed using Q and I^2^ statistics. We used a random-effects model for all the analyses due to the different type of statins and variety of dose of ginkgo biloba leaves used. Begg’s rank correlation test (Begg and Mazumdar, 1994) and Egger’s linear regression test (Egger et al., 1997) (*P* < 0.10 were considered to be statistically significant) were used to investigate the potential publication bias. Subgroup analysis was performed by the type of statins (atorvastatin vs. simvastatin) and treatment duration (≥8 vs. <8 weeks). All the statistical tests were done with RevMan 5.1 and STATA 11.0 with *P*-value of less than 0.05 was an indicator of statistical significance except for the heterogeneity test.

## Results

### Description of Studies

**FIGURE 1:**
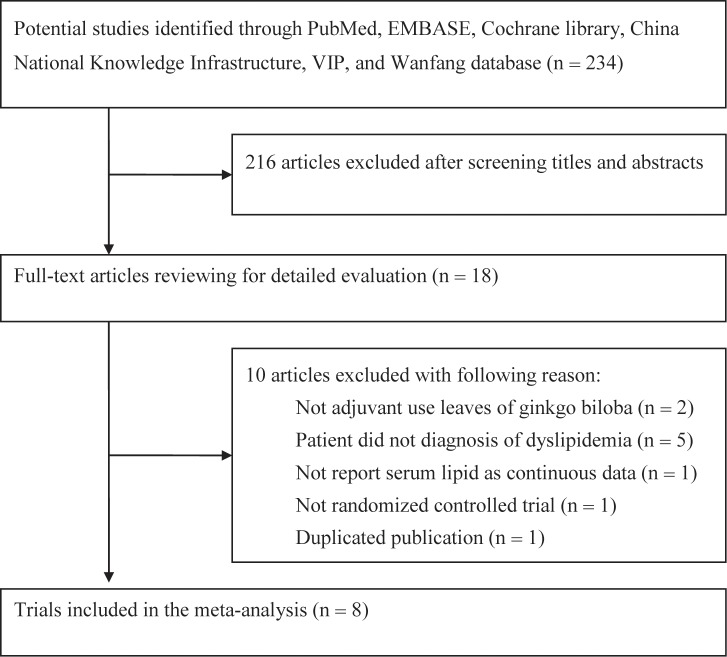
Flow chart showing study selection process.

**FIGURE 2:**
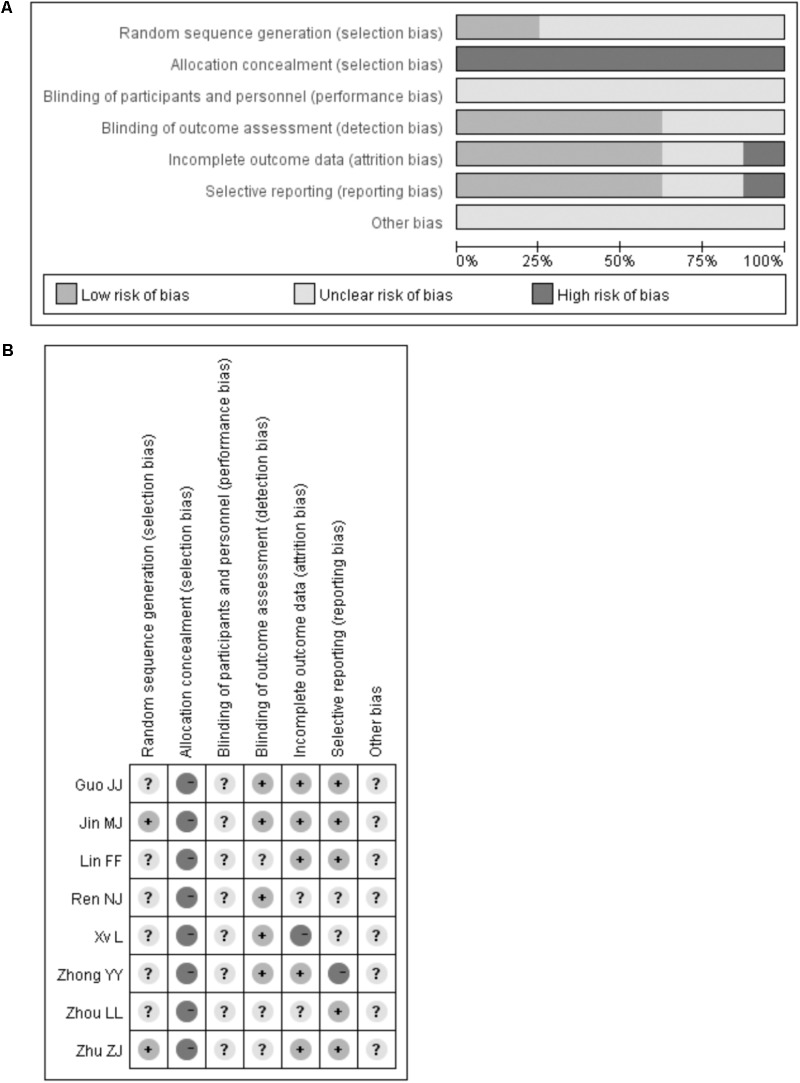
Quality assessment of the included trials. Risk of bias graph **(A)**. Risk of bias summary **(B)**.

A total of 234 potentially relevant studies were identified through our initial literature search. Of which, 216 articles were excluded after screening the title and abstract. After reviewing the full-text manuscript for eligibility, 8 RCTs (Zhou, 2010; Guo and Ju, 2011; Lin et al., 2011; Zhong and Bao, 2011; Jin et al., 2012; Ren, 2012; Zhu, 2012; Xu, 2013) met our predefined inclusion criteria. The study selection process is presented in **Figure 1**. The baseline characteristics of the included studies are shown in **Table 1**. Eight RCTs involving 664 patients were included, of which 334 cases received gingko biloba leaves plus statins and 330 cases received statins alone. All the studies were carried out in China and published between 2010 and 2013. The patient’s age ranged from 28 to 87 years. The available commercial gingko biloba leaves included tablet, capsule, and pill. The dose of gingko biloba leaves ranged from 120 to 576 mg per day. Atorvastatin was used in four trials, simvastatin was used in three trials, and one trial used rosuvastatin. Treatment duration ranged from 4 to 26 weeks. Using Cochrane risk of bias tool, the trials included in the analysis were generally judged to have low-quality with unclear risk of bias. The risk bias assessment of the included trials is presented in **Figure 2**.

**Table 1:** Summary of clinical trials included in meta-analysis.

Trial/year	Sample size	Mean age (men/women)	Patient entry criteria	Experiment group	Control group	Treatment duration	Outcome measures
Zhou, 2010	Exp:63	61–87	NP	Ginkgo biloba tablet 480 mg/d	Atorvastatin 20 mg/d	26 weeks	TG, TC, LDL-C, HDL-C
	Con:62	(66/59)	LDL-C ≥ 3.4 mmol/L, TG ≥ 1.7 mmol/L	LDL-C ≥ 3.4 mmol/L, TG ≥ 1.7 mmol/L			
Guo and Ju, 2011	Exp:40	46–79	TC ≥ 5.72 mmol,	Ginkgo biloba pill 480 mg/d +	Atorvastatin 20 mg/d	24 weeks	TC, LDL-C, HDL-C, AE
	Con:39	(44/36)	LDL-C ≥ 3.64 mmol/L, and/or HDL-C ≤ 1.04 mmol/L	atorvastatin 20 mg/d			
Zhong and Bao, 2011	Exp:30	39–78	TC ≥ 5.2 mmol,	Ginkgo biloba tablet 240 mg/d	Atorvastatin 10 mg/d	6 weeks	TG, TC, LDL-C, HDL-C
	Con:29	(29/30)	LDL-C ≥ 3.64 mmol/L, TG ≥ 1.7 mmol/L	+ atorvastatin 10 mg/d			
Lin et al., 2011	Exp:32	28–76	TC ≥ 5.2 mmol,	Ginkgo biloba tablet 240 mg/d	Atorvastatin 10 mg/d	6 weeks	TG, TC, LDL-C, HDL-C, AE
	Con:32	(35/29)	LDL-C ≥ 3.4 mmol/L, TG ≥ 1.7 mmol/L	+ atorvastatin 10 mg/d			
Jin et al., 2012	Exp:33	36–71	TC ≥ 5.2 mmol,	Ginkgo biloba tablet 240 mg/d	Rosuvastatin 10 mg/d	4 weeks	TG, TC, LDL-C, HDL-C, AE
	Con:32	(36/29)	LDL-C ≥ 3.64 mmol/L, TG ≥ 1.7 mmol/L	+ rosuvastatin 10 mg/d			
Ren, 2012	Exp:41	Exp:54.8 ± 3.2/(NP)	TC ≥ 5.2 mmol,	Ginkgo biloba tablet 576 mg/d	Simvastatin 20 mg/d	8 weeks	TG, TC, LDL-C, HDL-C
	Con:41	Con:59.1 ± 4.6/(NP)	LDL-C ≥ 3.4 mmol/L, TG ≥ 1.7 mmol/L	+ simvastatin 20 mg/d			
Zhu, 2012	Exp:65	42–78	TC ≥ 5.752 mmol,	Ginkgo biloba capsule	Simvastatin 10 mg/d	6 weeks	TG, TC, LDL-C, HDL-C
	Con:65	(62/58)	LDL-C ≥ 3.64 mmol/L, and/or HDL-C ≤ 1.04 mmol/L, TG ≥ 1.7 mmol/L	120 mg/d + simvastatin 20 mg/d			
Xu, 2013	Exp:30	47–80	TC ≥ 5.2 mmol,	Ginkgo biloba tablet	Simvastatin 20 mg/d	8 weeks	TG, TC, LDL-C, HDL-C
	Con:30	(37/23)	LDL-C ≥ 3.4 mmol/L, TG ≥ 1.7 mmol/L	240 mg/d + simvastatin 20 mg/d			


*Exp, experiment; Con, control; TGs, triglycerides; TC, total cholesterol; LDL-C, low density lipoprotein cholesterol; HDL-C, high-density lipoprotein cholesterol.*

### Serum Lipid Parameters

**FIGURE 3:**
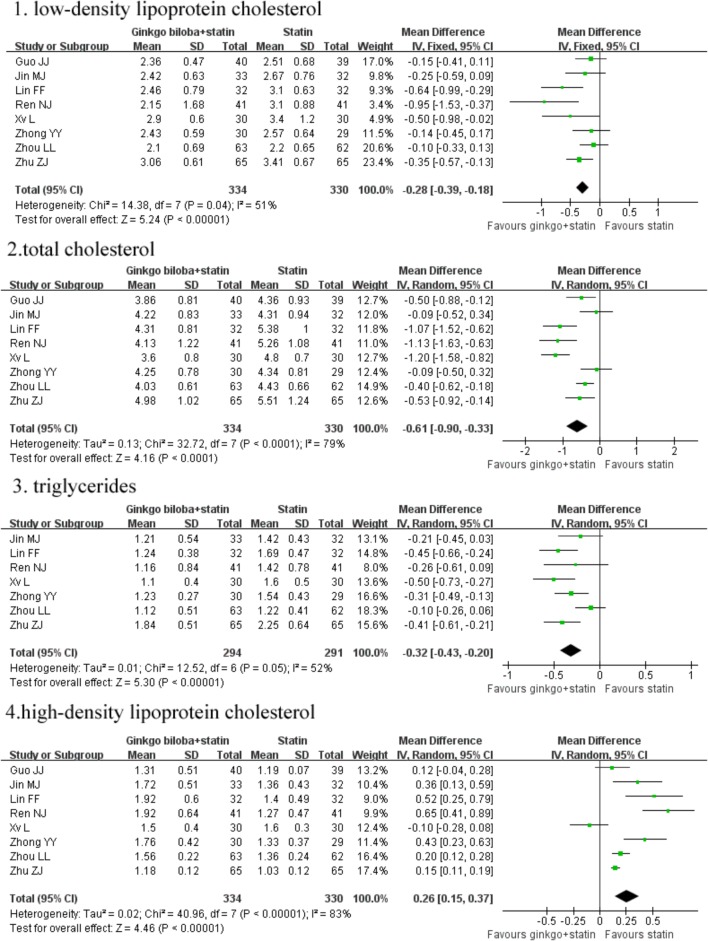
Forest plots of comparision of serum lipid parameters for ginkgo biloba leaves plus statins therapy versus statins therapy alone.

Eight trials (Zhou, 2010; Guo and Ju, 2011; Lin et al., 2011; Zhong and Bao, 2011; Jin et al., 2012; Ren, 2012; Zhu, 2012; Xu, 2013) reported serum LDL-C, HDL-C, and TC levels, and seven trials [15, 17–22] reported serum TG levels. As shown in **Figure 3**, compared with statin therapy alone, ginkgo biloba leaves in combination with statins therapy significantly reduced serum levels of TG (MD -0.32 mmol/L; 95% CI -0.43 to -0.20; *I*^2^ = 52.0%; *P* = 0.05), TC (MD -0.61 mmol/L; 95% CI -0.90 to -0.33; *I*^2^ = 79.0%; *P* < 0.001), LDL-C (MD -0.32 mmol/L; 95% CI -0.48 to -0.16; *I*^2^ = 51.0%; *P* = 0.04), and increased serum level of HDL-C (MD 0.26 mmol/L; 95% CI: 0.15 to 0.37; *I*^2^ = 83%; *P* < 0.001).

### Subgroup Analyses and Publication Bias

We conducted a subgroup analysis based on the type of statins. The detailed findings of subgroup analysis are presented in **Table 2**. Ginkgo biloba leaves plus simvastatin therapy appeared to achieve a greater improvement in serum levels of TG, TC, and LDL-C than in combination with atorvastatin therapy. Ginkgo biloba leaves plus atorvastatin therapy achieved a greater increment in serum HDL-C levels. We did not conducted subgroup analysis based on the dose of ginkgo biloba leaves and duration of treatment mainly due to the significant heterogeneity. No significant publication bias was observed serum levels of LDL-C (*P*_Begg_ = 0.902 and *P*_Egger_ = 0.401), HDL-C (*P*_Begg_ = 0.902 and *P*_Egger_ = 0.401), TG (*P*_Begg_ = 1.000 and *P*_Egger_ = 0.431) and TC (*P*_Begg_ = 0.902 and *P*_Egger_ = 0.401).

**Table 2:** Subgroup analyses on the changes of serum lipids level.

Subgroups	Number of trials	Pooled weighted mean difference	95% confidence interval	Heterogeneity between trials
**(1) LDL-C (mmol/L)**			
**Type of statins**				
Atorvastatin	4	-0.23	-0.45 to -0.22	*P* < 0.071; *I*^2^ = 57.4%
Simvastatin	3	-0.52	-0.83 to -0.20	*P* = 0.160; *I*^2^ = 45.4%
**Treatment duration**				
≥8 weeks	4	-0.33	-0.63 to -0.03	*P* = 0.034; *I*^2^ = 65.4%
<8 weeks	4	-0.34	-0.52 to -0.15	*P* = 0.199; *I*^2^ = 35.5%
**(2) HDL-C (mmol/L)**			
**Type of statins**				
Atorvastatin	4	0.29	0.13 to 0.44	*P* = 0.014; *I*^2^ = 71.7%
Simvastatin	3	0.22	-0.08 to 0.52	*P* < 0.001; *I*^2^ = 91.6%
**Treatment duration**				
≥8 weeks	4	0.20	-0.02 to 0.42	*P* < 0.001; *I*^2^ = 87.8%
<8 weeks	4	0.34	0.14 to 0.54	*P* = 0.001; *I*^2^ = 81.7%
**(3) TC (mmol/L)**			
**Type of statins**				
Atorvastatin	4	-0.50	-0.83 to -0.16	*P* = 0.013; *I*^2^ = 72.0%
Simvastatin	3	-0.95	-1.39 to -0.51	*P* = 0.037; *I*^2^ = 69.6%
**Treatment duration**				
≥8 weeks	4	-0.78	-1.21 to -0.36	*P* = 0.001; *I*^2^ = 82.3%
<8 weeks	4	-0.44	-0.88 to -0.00	*P* = 0.004; *I*^2^ = 77.4%
**(4) TG (mmol/L)**				
**Type of statins**				
Atorvastatin	3	-0.28	-0.48 to -0.08	*P* = 0.028; *I*^2^ = 72.2%
Simvastatin	3	-0.42	-0.56 to -0.28	*P* = 0.528; *I*^2^ = 0.0%
**Treatment duration**				
≥8 weeks	4	-0.28	-0.55 to -0.01	*P* = 0.020; *I*^2^ = 74.4%
<8 weeks	4	-0.35	-0.45 to -0.25	*P* = 0.432; *I*^2^ = 0.0%


### Adverse Events

Three trials (Guo and Ju, 2011; Lin et al., 2011; Jin et al., 2012) reported adverse events. Lin et al. (2011) study did not observe any adverse events and without any patients dropout. In Jin et al. (2012) study elevated serum levels of aminotransferase were observed in two cases in the control group and one case in the experimental group. In addition, one case developed gastrointestinal tract reactions in the experimental group. Serum creatinine and urea nitrogen levels were normal in all the patients. In Guo and Ju (2011) study, one case was dropped out in the control group and no significant changes in the value of blood routine, liver and kidney function, creatine kinase tests.

## Discussion

Our meta-analysis suggests that ginkgo biloba leaves in combination with statins therapy significantly improve serum levels of TC (0.61 mmol/L), LDL-C (0.32 mmol/L), HDL-C (0.26 mmol/L), and TG (0.32 mmol/L) compared with statins therapy alone in patients with dyslipidemia. Combination of ginkgo biloba leaves with statins therapy may achieve greater improvement in serum lipids for patients with the mixed dyslipidemia. However, whether concomitant treatment with each statin and ginkgo biloba leaves increases or reduces adverse events are largely unknown. Atorvastatin, rosuvastatin, and simvastatin were used in the current study. Subgroup analyses indicated that ginkgo biloba leaves plus simvastatin were associated with a greater reduction in serum levels of TG, TC, and LDL-C. While the combination of ginkgo biloba leaves with atorvastatin therapy achieved a greater increase serum HDL-C levels. This difference needs to be further studied because of differential metabolic effects of distinct statins (Koh et al., 2011).

In China, herbal formula alone or in combination with statins is common in management of dyslipidemia. Ginkgo biloba leaves alone also showed beneficial effects on patients with hyperlipidemia (Sun et al., 2001) and hypercholestrolemia in children with nephrotic syndrome (Zhong et al., 2007). Experimental studies showed that pretreatment with ginkgo biloba leaves extract prevented hyperlipidemia in rat (Yao et al., 2007; Zhong and Li, 2009; Wu, 2013; Banin et al., 2014) and quail models (Wei et al., 2003). Contrary to our findings, Ginkgo biloba leaves extract 360 mg GBE daily for 14 days had no significant effects on cholesterol-lowering efficacy of atorvastatin on healthy volunteers (Guo et al., 2012).

An important concern is the potential adverse effects of ginkgo biloba leaves. However, only three included trials (Guo and Ju, 2011; Lin et al., 2011; Jin et al., 2012) reported the adverse events. The common adverse event of ginkgo biloba leaves was gastrointestinal disturbances. No serious adverse events were reported in the included trials. These findings indicated that the administration of ginkgo biloba leaves appeared to be relatively safe up to 26 weeks’ treatment. The frequently reported adverse events in the literatures included gastrointestinal disturbances, dizziness, headaches, nausea, diarrhea, excessive bleeding, insomnia, stress ulcer, allergic skin reactions, etc. (Yang et al., 2014). Administration of ginkgo biloba may prolong bleeding times because of its anti-platelet properties (Dugoua et al., 2006). Therefore, ginkgo biloba should be administered with caution during pregnancy and lactation.

The mechanism of lipid modifying effects with ginkgo biloba leaves has not been fully elucidated. Ginkgo biloba leaves have been used to treat hyperlipidemia patients with concurrent coronary heart disease or cerebrovascular diseases. The cardiovascular protective effects of ginkgo biloba leaves can be attributed to the following aspects: antioxidant properties, anti-platelet activity, and increased blood flow through the release of nitric oxide (Mahady, 2002; Zhou et al., 2004). Hepatic toxicity is one of common adverse effects due to statins. For the target lipid-lowering, adjuvant treatment with ginkgo biloba leaves to statins therapy might reduce the dose of statins. Thus, concomitant treatment with statins and ginkgo biloba leaves may result in a reduction of hepatic toxicity of statins. This assumption was supported by a high dose of ginkgo biloba leaves could slightly decrease the plasma statins concentrations (Liu et al., 2009; Guo et al., 2012).

There are some limitations in this study. First, most of the included trials had unclear risk of bias according to the Cochrane risk of bias tool; it is too early to reach a reliable conclusion. Second, patients enrollment did not consider the TCM diagnosis and syndromes differentiation; potential selection bias may lower the efficacy of ginkgo biloba leaves treatment. Third, we had insufficient data to conduct subgroup analysis based on regimens of ginkgo biloba leaves and patients’ characteristics. Fourth, treatment duration was very different (from 4 to 26 weeks) and the dose of ginkgo biloba leaves was highly variable (from 120 to 576 mg/day). Therefore, this meta-analysis could not establish the appropriate dose and treatment duration of ginkgo biloba leaves. Fifth, future well-designed studies with large sample size are needed to confirm our findings because the studied population was limited to 664 patients in the analysis. Finally, significant heterogeneity was found in the pooling serum lipid parameters, which might be attributed to the different regimen of ginkgo biloba leaves and statins.

## Conclusion

This meta-analysis suggests that adjuvant treatment with ginkgo biloba leaves appears to have incremental benefits in the management of serum lipids than statins therapy alone. More well-designed RCTs are needed to investigate the benefits of the combination of statins and ginkgo biloba leaves due to the methodological flaws of the included trials. Moreover, whether adding ginkgo biloba leaves to statins has additional cardiovascular risk reduction needs to be further investigated.

## Author Contributions

YF and XJ made the literature search, extracted data, and evaluated the study quality. CM performed the statistical analysis. YF drafted the manuscript. DG designed the study, interpreted the results, and revised the manuscript.

## Conflict of Interest Statement

The authors declare that the research was conducted in the absence of any commercial or financial relationships that could be construed as a potential conflict of interest.
